# Supplementary material for: Optimizing target nodes selection for the control energy of directed complex networks
Source: Sci Rep. 2020 Oct 22;10:18112. doi: 10.1038/s41598-020-75101-w (PMC7581767; doi:10.1038/s41598-020-75101-w)
Supplement: Supplementary file 1 — Supplementary Information. [file 41598_2020_75101_MOESM1_ESM.pdf]

Optimizing target nodes selection for the control energy of directed complex networks
Supplementary Information

Hong Chen[1] and Ee Hou Yong[1,*]

[1]Division of Physics and Applied Physics, School of Physical and Mathematical Sciences, Nanyang Technological University, Singapore, 637371, Singapore.
[*]Correspondence and requests for materials should be addressed to E.H.Y. (email: eehou@ntu.edu.sg)

Contents

1 Introduction **2**
1.1 Controllability Gramian Definition . . . 2
1.2 Trace properties . . . 2
1.3 Review of probability theory standard result . . . 2

2 Review of Matrix Derivative and Index Notation **2**
2.1 Derivative of Inverse . . . 3
2.2 Chain Rule . . . 3
2.3 Product Rule . . . 4

3 Energy cost function **4**

4 Energy gradient information **6**

5 Selecting C^*_{binary} from C^* **10**

6 Dataset **11**
6.1 Main text table 1 additional information . . . 11

1 Introduction

In this supplement, we will show how to derive the cost function and the cost function gradient information in the main text. In sections 1 and 2, we review the mathematical tools needed for the derivation. In section 3, we derive the energy cost function. In section 4, we derive the energy cost function gradient information. We also explain how to obtain C^*_{binary} from C^* in section 5. Finally, we discuss network data set as well as provide additional information with regards to standard deviations of table 1 of the main text in section 6.

1.1 Controllability Gramian Definition

The controllability Gramian matrix $W = \int_0^{t_f} e^{A(t_f-t)}BB^T e^{A^T(t_f-t)}dt$ is symmetric. Thus, for Gramian index notation, $W_{ij} = W_{ji}$.

Likewise for output controllability Gramian $U = (CWC^T)$, it is symmetric. Thus, the inverse of output controllability Gramian is also symmetric: $[U^{-1}]^T = U^{-1}$.

1.2 Trace properties

We can write the trace of a matrix index notation as: $tr(A) = \sum_a A_{aa} = A_{aa}$. And for trace of matrices multiplication:

$$tr(ABC) = \sum_a \sum_b \sum_c A_{ab}B_{bc}C_{ca} = A_{ab}B_{bc}C_{ca} \tag{S 1}$$

Where the indices were chosen arbitrarily and the summation operator is dropped for compactness. For arbitrary length of matrices multiplication, the index notation rule holds analogously.

1.3 Review of probability theory standard result

Suppose we have a random variable x, distributed with mean zero and standard deviation 1: $x \sim \mathcal{N}(0, 1)$.

Then a vector $\mathbf{x}$, where $\mathbf{x} \in \mathbb{R}^{N\times 1}$, which contains random variable x as its vector element is also distributed as standard normal distribution: $\mathbf{x} \sim \mathcal{N}(0, 1)$.

Since mean is zero, the expectation value of this vector is zero: $\mathbb{E}(\mathbf{x}_0) = [0, \, ... \, , 0]^T$.

For the expectation value of the covariance matrix of $\mathbf{x}$, a standard result in probability theory is given as: $\mathbb{E}(\mathbf{x}_0\mathbf{x}_0^T) = I_N$.

Where I_N is an $N \times N$ identity matrix.

2 Review of Matrix Derivative and Index Notation

In this section, we review Matrix Derivatives and Index Notation, which we need to solve for the cost function matrix derivative gradient information. Many of the results are obtained with the help of the matrix cookbook [S1], and some supporting lemmas from [S2].

Basic Derivative. For arbitrary matrix element X, the derivative of X with itself X_{ij} is given as:

$$\frac{\partial X_{kl}}{\partial X_{ij}} = \delta_{ki} \cdot \delta_{lj} = \delta_{lj} \cdot \delta_{ki}, \tag{S 2}$$

And the matrix transpose derivative with itself:

$$\frac{\partial X^T_{kl}}{\partial X_{ij}} = \delta_{kj} \cdot \delta_{li} = \delta_{li} \cdot \delta_{kj}. \tag{S 3}$$

Where the subscripts $_{kl}$ and $_{ij}$ are used to identify the matrix elements of each of the matrix, and $\delta_{ij} = 1$ iff $i = j$, $\delta_{ij} = 0$ otherwise. The kronecker delta terms δ_{ij} is basically the same as matrix elements of an identity matrix.

Transpose index notation.

$$X^T_{ij} = X_{ji}. \tag{S 4}$$

Dealing with index notation delta terms.

$$\delta_{ki} \cdot \delta_{ij} = \delta_{kj} \tag{S 5}$$

For arbitrary matrix element X_{ij},

$$X_{ij} \cdot \delta_{ja} = X_{ia} \tag{S 6}$$

$$\delta_{ia} \cdot X_{ij} = [X^T]_{ji} \cdot \delta_{ia} = [X^T]_{ja} = X_{aj} \tag{S 7}$$

Compatible matrices. Suppose $X = AB$, then matrix element X_{ij}:

$$X_{ij} = \sum_k A_{ik}B_{kj} = A_{ik} \cdot B_{kj}, \tag{S 8}$$

and likewise for compatible matrices $X = ABC$, then matrix element X_{ij}:

$$X_{ij} = [ABC]_{ij} = \sum_k \sum_p [A]_{ak} \cdot [B]_{kp} \cdot [C]_{pj} = [A]_{ak} \cdot [B]_{kp} \cdot [C]_{pj}. \tag{S 9}$$

Where in (S 8) and (S 9), we have dropped the summation notation for simplicity and compactness.

2.1 Derivative of Inverse

For an invertible matrix X, the derivative of the inverse of X with itself is given by:

$$\frac{\partial [X^{-1}]_{kl}}{\partial [X]_{ij}} = -[X^{-1}]_{ki}[X^{-1}]_{jl}. \tag{S 10}$$

2.2 Chain Rule

Suppose we are interested in matrix derivative of a function of matrix, with respect to matrix A, we can use chain rule as follows:

$$\frac{\partial f(A, U(A))}{\partial A} = \frac{\partial f(A, U(A))}{\partial U} \cdot \frac{\partial U(A)}{\partial A}. \tag{S 11}$$

Where $f(\cdot)$ is a function of matrix A and matrix function $U(A)$, where matrix function $U(A)$ is a function of matrix A.

2.3 Product Rule

Suppose we have an arbitrary length of matrix and matrix function multiplication, and we want to perform the matrix derivative with respect to matrix A, we can apply the product rule as follows:

$$\begin{aligned}\frac{\partial}{\partial A}[A\cdot f(A)\cdot g(B)\cdot B\cdot A] &= \frac{\partial A}{\partial A}\cdot[f(A)\cdot g(B)\cdot B\cdot A]\\ &+ A\cdot\frac{\partial f(A)}{\partial A}\cdot[g(B)\cdot B\cdot A]\\ &+ [A\cdot f(A)\cdot g(B)\cdot B]\cdot\frac{\partial A}{\partial A}\end{aligned} \tag{S 12}$$

where $f(A)$ is a matrix function of matrix A, and $g(B)$ is a matrix function of matrix B. For arbitrary length of matrices multiplication, product rule holds analogously.

3 Energy cost function

We want to derive the energy cost function, which is the expected value of the energy cost function over all realisations of the initial state vector $\mathbf{x}_0$, picked from standard normal distribution (that is, $\mathbf{x}_0 \sim \mathcal{N}(0, 1)$),

$$\mathcal{E}(C^T) = \mathbb{E}[\int_0^{t_f} \mathbf{u}^T(t)\mathbf{u}(t)dt]. \tag{S 13}$$

From [S3], energy-optimal control signal to target control subset P number of nodes is derived from optimal control theory [S4] as:

$$\mathbf{u}^*(t) = B^T e^{A^T(t_f-t)} C^T (CWC^T)^{-1}(\mathbf{y}_f - Ce^{A(t_f-t_0)}\mathbf{x}_0). \tag{S 14}$$

As a review, we recall from the main text the matrix dimensions: $A \in \mathbb{R}^{N\times N}$, $B \in \mathbb{R}^{N\times M}$, $C \in \mathbb{R}^{P\times N}$, $\mathbf{x}(t)\in\mathbb{R}^{N\times 1}$, $\mathbf{y}(t)\in\mathbb{R}^{P\times 1}$, $\mathbf{u}(t)\in\mathbb{R}^{M\times 1}$.

Expanding (S 14) and setting $t_0 = 0$ for simplicity we obtain:

$$\mathbf{u}^*(t) = B^T e^{A^T(t_f-t)} C^T (CWC^T)^{-1}\mathbf{y}_f - B^T e^{A^T(t_f-t)} C^T (CWC^T)^{-1} Ce^{At_f}\mathbf{x}_0. \tag{S 15}$$

It is then straight-forward to obtain its transpose:

$$\mathbf{u}^{*T}(t) = \mathbf{y}_f^T (CWC^T)^{-1} Ce^{A(t_f-t)}B - \mathbf{x}_0^T e^{A^T t_f} C^T (CWC^T)^{-1} Ce^{A(t_f-t)}B. \tag{S 16}$$

Note that since the $P \times P$ output controllability matrix (CWC^T) is symmetric, real, and non-negative definite [S3], then transpose operation has no effect on it, nor its inverse: that is, $[(CWC^T)^{-1}]^T = (CWC^T)^{-1}$.

Next, we evaluate $\mathbf{u}^{*T}(t)\mathbf{u}^*(t)$, as a preamble to (S 13).

$$
\begin{aligned}
\mathbf{u}^{*T}(t)\mathbf{u}^{*}(t) =& [\mathbf{y}_f^T(CWC^T)^{-1}Ce^{A(t_f-t)}B - \mathbf{x}_0^T e^{A^T t_f}C^T(CWC^T)^{-1}Ce^{A(t_f-t)}B] \\
&\times [B^T e^{A^T(t_f-t)}C^T(CWC^T)^{-1}\mathbf{y}_f - B^T e^{A^T(t_f-t)}C^T(CWC^T)^{-1}Ce^{At_f}\mathbf{x}_0] \\
=& \mathbf{y}_f^T(CWC^T)^{-1}Ce^{A(t_f-t)}BB^T e^{A^T(t_f-t)}C^T(CWC^T)^{-1}\mathbf{y}_f \\
&- \mathbf{x}_0^T e^{A^T t_f}C^T(CWC^T)^{-1}Ce^{A(t_f-t)}BB^T e^{A^T(t_f-t)}C^T(CWC^T)^{-1}\mathbf{y}_f \\
&- \mathbf{y}_f^T(CWC^T)^{-1}Ce^{A(t_f-t)}BB^T e^{A^T(t_f-t)}C^T(CWC^T)^{-1}Ce^{At_f}\mathbf{x}_0 \\
&+ \mathbf{x}_0^T e^{A^T t_f}C^T(CWC^T)^{-1}Ce^{A(t_f-t)}BB^T e^{A^T(t_f-t)}C^T(CWC^T)^{-1}Ce^{At_f}\mathbf{x}_0.
\end{aligned}
\tag{S 17}
$$

Substituting (S 17) into (S 13), we get:

$$
\begin{aligned}
&\mathcal{E}(C^T) = \\
&\mathbb{E}\Bigg[\int_0^{t_f} \mathbf{y}_f^T(CWC^T)^{-1}Ce^{A(t_f-t)}BB^T e^{A^T(t_f-t)}C^T(CWC^T)^{-1}\mathbf{y}_f \\
&- \mathbf{x}_0^T e^{A^T t_f}C^T(CWC^T)^{-1}Ce^{A(t_f-t)}BB^T e^{A^T(t_f-t)}C^T(CWC^T)^{-1}\mathbf{y}_f \\
&- \mathbf{y}_f^T(CWC^T)^{-1}Ce^{A(t_f-t)}BB^T e^{A^T(t_f-t)}C^T(CWC^T)^{-1}Ce^{At_f}\mathbf{x}_0 \\
&+ \mathbf{x}_0^T e^{A^T t_f}C^T(CWC^T)^{-1}Ce^{A(t_f-t)}BB^T e^{A^T(t_f-t)}C^T(CWC^T)^{-1}Ce^{At_f}\mathbf{x}_0 \, dt\Bigg].
\end{aligned}
\tag{S 18}
$$

We note the dimensions of each of the terms. The first term $[\mathbf{y}_f^T \ldots \mathbf{y}_f]$ has dimensionality 1×1 since $\mathbf{y}^T \in \mathbb{R}^{1\times P}$ and $\mathbf{y} \in \mathbb{R}^{P\times 1}$. Likewise, the second term $[\mathbf{x}_0^T \ldots \mathbf{y}_f]$, third term $[\mathbf{y}_f^T \ldots \mathbf{x}_0]$, and fourth term $[\mathbf{x}_0^T \ldots \mathbf{x}_0]$ all have 1×1 dimensionality.

Thus, we can apply the trace operator to (S 18):

$$
\begin{aligned}
&\mathcal{E}(C^T) = \\
&\mathbb{E}\Bigg[\int_0^{t_f} tr\Big(\mathbf{y}_f^T(CWC^T)^{-1}Ce^{A(t_f-t)}BB^T e^{A^T(t_f-t)}C^T(CWC^T)^{-1}\mathbf{y}_f\Big) \\
&- tr\Big(\mathbf{x}_0^T e^{A^T t_f}C^T(CWC^T)^{-1}Ce^{A(t_f-t)}BB^T e^{A^T(t_f-t)}C^T(CWC^T)^{-1}\mathbf{y}_f\Big) \\
&- tr\Big(\mathbf{y}_f^T(CWC^T)^{-1}Ce^{A(t_f-t)}BB^T e^{A^T(t_f-t)}C^T(CWC^T)^{-1}Ce^{At_f}\mathbf{x}_0\Big) \\
&+ tr\Big(\mathbf{x}_0^T e^{A^T t_f}C^T(CWC^T)^{-1}Ce^{A(t_f-t)}BB^T e^{A^T(t_f-t)}C^T(CWC^T)^{-1}Ce^{At_f}\mathbf{x}_0\Big) \, dt\Bigg]
\end{aligned}
\tag{S 19}
$$

$$
\begin{aligned}
=& \int_0^{t_f} tr\Big(\mathbf{y}_f^T(CWC^T)^{-1}Ce^{A(t_f-t)}BB^T e^{A^T(t_f-t)}C^T(CWC^T)^{-1}\mathbf{y}_f\Big)\, dt \\
& \int_0^{t_f} -tr\Big(\cancel{\mathbb{E}[\mathbf{x}_0^T]e^{A^T t_f}C^T(CWC^T)^{-1}Ce^{A(t_f-t)}BB^T e^{A^T(t_f-t)}C^T(CWC^T)^{-1}\mathbf{y}_f}\Big) dt \\
& \int_0^{t_f} -tr\Big(\cancel{\mathbf{y}_f^T(CWC^T)^{-1}Ce^{A(t_f-t)}BB^T e^{A^T(t_f-t)}C^T(CWC^T)^{-1}Ce^{At_f}\mathbb{E}[\mathbf{x}_0]}\Big)\, dt \\
& \int_0^{t_f} +tr\Big(e^{A^T t_f}C^T(CWC^T)^{-1}Ce^{A(t_f-t)}BB^T e^{A^T(t_f-t)}C^T(CWC^T)^{-1}Ce^{At_f}\mathbb{E}[\mathbf{x}_0\mathbf{x}_0^T]\Big)\, dt
\end{aligned}
\tag{S 20}
$$

$$
\begin{aligned}
&= tr\bigg(\mathbf{y}_f^T(CWC^T)^{-1}C\bigg[\int_0^{t_f} e^{A(t_f-t)}BB^Te^{A^T(t_f-t)}dt\bigg]C^T(CWC^T)^{-1}\mathbf{y}_f\bigg) \\
&+ tr\bigg(e^{A^Tt_f}C^T(CWC^T)^{-1}C\int_0^{t_f}\bigg[e^{A(t_f-t)}BB^Te^{A^T(t_f-t)}dt\bigg]C^T(CWC^T)^{-1}Ce^{At_f}\bigg)
\end{aligned} \tag{S 21}
$$

$$
\begin{aligned}
&= tr\bigg(\mathbf{y}_f^T(CWC^T)^{-1}CWC^T(CWC^T)^{-1}\mathbf{y}_f\bigg) \\
&+ tr\bigg(e^{A^Tt_f}C^T(CWC^T)^{-1}CWC^T(CWC^T)^{-1}Ce^{At_f}\bigg)
\end{aligned} \tag{S 22}
$$

$$
\therefore \mathcal{E}(C^T) = tr\bigg((CWC^T)^{-1}\mathbf{y}_f\mathbf{y}_f^T\bigg) + tr\bigg(C^T(CWC^T)^{-1}Ce^{At_f}e^{A^Tt_f}\bigg). \tag{S 23}
$$

Where in (S 20), the expectation operator $\mathbb{E}[\cdot]$ only acts on random variable matrix, which are mainly $\mathbf{x}_0$ or $\mathbf{x}_0^T$ only. Since the first term does not contain any random variable matrix, the expectation operator does not operate on it. Because $\mathbf{x}_0 \sim \mathcal{N}(0,1)$, then $\mathbb{E}[\mathbf{x}_0] = [0,\ \dots,0]^T$, and thus the second and third terms of matrix multiplication becomes zero. Similarly, $\mathbb{E}[\mathbf{x}_0\mathbf{x}_0^T] = I_N$, where I_N is an $N \times N$ identity matrix, which is a standard result in probability theory. In the third term, we moved the matrix $\mathbf{x}_0^T$ to the end using the cyclic permutation property of trace.

In (S 21), the integration operator only acts on time-dependent terms, while the non time-dependent terms are treated as constants, thus we can move the integration operator within the trace. We observe that the term $[\int_0^{t_f} e^{A(t_f-t)}BB^Te^{A^T(t_f-t)}dt] = W$ is the definition of the controllability Gramian, as mentioned in the main text.

In (S 22), we observe that the terms $(CWC^T)^{-1}(CWC^T) = I_P$, where I_P is a $P \times P$ identity matrix, since the matrices in the left parenthesis is just the inverse of the matrices in the right parenthesis. And in (S 23), we move the terms around using trace cyclic permutation to concatenate the terms which are not related to matrix C for ease of derivation of the energy gradient information.

Thus, the energy cost function is derived.

4 Energy gradient information

To begin, we first re-write the energy cost function, equation (S 23) as:

$$
\mathcal{E}(C^T) = tr\bigg((CWC^T)^{-1}\mathbf{Y}_{f\prime}\bigg) + tr\bigg(C^T(CWC^T)^{-1}C\mathbf{X}_{f\prime}\bigg) \tag{S 24}
$$

for convenience. Where $\mathbf{Y}_{f\prime} = \mathbf{y}_f\mathbf{y}_f^T$ and $\mathbf{X}_{f\prime} = e^{At_f}e^{A^Tt_f}$ are treated as constant terms which do not contain any matrix C dependence.

Next, we write out the trace terms of (S 24) as summation with index notation form as it is easier to deal with,

$$\begin{aligned}\mathcal{E}(C^T) &= \sum_a \sum_b [CWC^T]^{-1}_{ab}[\mathbf{Y}_{f\prime}]_{ba} + \sum_c \sum_d \sum_e \sum_f [C^T]_{cd}[(CWC^T)^{-1}]_{de}[C]_{ef}[\mathbf{X}_{f\prime}]_{fc} \\ &= \sum_a \sum_b [U]^{-1}_{ab}[\mathbf{Y}_{f\prime}]_{ba} + \sum_c \sum_d \sum_e \sum_f [C^T]_{cd}[U^{-1}]_{de}[C]_{ef}[\mathbf{X}_{f\prime}]_{fc} \\ &= [U]^{-1}_{ab}[\mathbf{Y}_{f\prime}]_{ba} + [C^T]_{cd}[U^{-1}]_{de}[C]_{ef}[\mathbf{X}_{f\prime}]_{fc}.\end{aligned} \tag{S 25}$$

Where we have treated the output controllability Gramian term $(CWC^T) = U$ and dropped the summation operator for compactness (see (S 1)).

We are interested in deriving the energy gradient, $\dfrac{\partial \mathcal{E}(C^T)}{\partial C^T}$:

$$\begin{aligned}\frac{\partial \mathcal{E}(C^T)}{\partial [C^T]_{NP}} &= \frac{\partial}{\partial [C^T]_{NP}}\Big\{[U]^{-1}_{ab}[\mathbf{Y}_{f\prime}]_{ba}\Big\} \\ &+ \frac{\partial}{\partial [C^T]_{NP}}\Big\{[C^T]_{cd}[U^{-1}]_{de}[C]_{ef}[\mathbf{X}_{f\prime}]_{fc}\Big\},\end{aligned} \tag{S 26}$$

which is the derivative of the energy cost function with respect to matrix C^T. Note that the subscript $_{NP}$ appended to the matrix derivative term, $[C^T]_{NP}$ indicates the matrix derivative term index notation, which represents an arbitrary matrix element of the gradient information matrix.

Next,

$$\begin{aligned}\frac{\partial \mathcal{E}(C^T)}{\partial [C^T]_{NP}} &= \frac{\partial [U^-1]_{ab}}{\partial [U]_{gh}}\frac{\partial [U]_{gh}}{\partial [C^T]_{NP}}[\mathbf{Y}_{f\prime}]_{ba} \\ &+ \Bigg[\frac{\partial [C^T]_{cd}}{\partial [C^T]_{NP}}[U^{-1}]_{de}[C]_{ef}[\mathbf{X}_{f\prime}]_{fc} \\ &+ [C^T]_{cd}\Bigg\{\frac{\partial [U^{-1}]_{de}}{\partial [U]_{gh}}\frac{\partial [U]_{gh}}{\partial [C^T]_{NP}}\Bigg\}[C]_{ef}[\mathbf{X}_{f\prime}]_{fc} \\ &+ [C^T]_{cd}[U^{-1}]_{de}\frac{\partial [C]_{ef}}{\partial [C^T]_{NP}}[X_{f\prime}]_{fc}\Bigg].\end{aligned} \tag{S 27}$$

Where we have applied both chain rule to deal with the inverse term $[U^{-1}]$ and product rule to deal with matrix multiplication terms with matrix dependence on matrix C^T (see equations (S 11) and (S 12)).

At this juncture, we take a detour to solve the chain rule part of (S 27), which we will substitute back.

In particular, we are interested to solve $\dfrac{\partial [U^{-1}]_{ab}}{\partial [U]_{gh}}\left(\dfrac{\partial [U^{-1}]_{de}}{\partial [U]_{gh}}\right)$ and $\dfrac{\partial [U]_{gh}}{\partial [C^T]_{NP}}$.

To begin,

$$\frac{\partial [U^{-1}]_{ab}}{\partial [U]_{gh}} = -[U^{-1}]_{ag}[U^{-1}]_{hb}. \tag{S 28}$$

$$\frac{\partial [U^{-1}]_{de}}{\partial [U]_{gh}} = -[U^{-1}]_{dg}[U^{-1}]_{he}. \tag{S 29}$$

Where we have used equation (S 10) from **Review of Matrix Derivative and Index Notation**.

Next, we wish to solve $\frac{\partial [U]_{gh}}{\partial [C^T]_{NP}}$, where we recall that we have denoted earlier $U = (CWC^T)$. Thus,

$$\frac{\partial [U]_{gh}}{\partial [C^T]_{NP}} = \frac{\partial}{\partial [C^T]_{NP}} \cdot [CWC^T]_{gh} \tag{S 30}$$

$$= \frac{\partial}{\partial [C^T]_{NP}} \Big\{ [C]_{gi} [W]_{ij} [C^T]_{jh} \Big\} \tag{S 31}$$

$$= \left[\frac{\partial [C]_{gi}}{\partial [C^T]_{NP}} \right] [W]_{ij} [C^T]_{jh} + [C]_{gi} [W]_{ij} \left[\frac{\partial [C^T]_{jh}}{\partial [C^T]_{NP}} \right] \tag{S 32}$$

$$Denote\ [C^T] = [\hat{C}]\ and\ [C] = [\hat{C}^T]$$

$$= \left[\frac{\partial [\hat{C}^T]_{gi}}{\partial [\hat{C}]_{NP}} \right] [W]_{ij} [\hat{C}]_{jh} + [\hat{C}^T]_{gi} [W]_{ij} \left[\frac{\partial [\hat{C}]_{jh}}{\partial [\hat{C}]_{NP}} \right] \tag{S 33}$$

$$\therefore \frac{\partial [U]_{gh}}{\partial [C^T]_{NP}} = \delta_{gP} \delta_{iN} [W]_{ij} [C^T]_{jh} + [C]_{gi} [W]_{ij} \delta_{jN} \delta_{hP}. \tag{S 34}$$

In (S 31), we used (S 9) to write out the matrix multiplication terms as separate terms with index notation. The indices i and j were chosen arbitrarily.

In (S 32), we apply product rule. Since the controllability Gramian W does not have C matrix dependence, it is treated as a constant with respect to matrix C.

In (S 33), we write C^T as $\hat{C}$, and C as $\hat{C}^T$. Then, we apply the basic derivative formula (S 2) and (S 3).

Finally, in (S 34), we obtain the solution and undo the overhat notation of previous step. (S 34) will be used to derive the energy gradient when we substitute it back.

Substituting the chain rule part of the result, (S 28), (S 29) and (S 34), into the energy gradient, (S 27), we obtain:

$$\begin{aligned}
&\frac{\partial \mathcal{E}(C^T)}{\partial [C^T]_{NP}} \\
&\quad = - [U^{-1}]_{ag} [U^{-1}]_{hb} \Big[\delta_{gP} \delta_{iN} [W]_{ij} [C^T]_{jh} + [C]_{gi} [W]_{ij} \delta_{jN} \delta_{hP} \Big] [\mathbf{Y}_{f\prime}]_{ba} \\
&\quad + \Bigg[\delta_{cN} \delta_{dP} [U^{-1}]_{de} [C]_{ef} [\mathbf{X}_{f\prime}]_{fc} - [C^T]_{cd} \Big\{ [U^{-1}]_{dg} [U^{-1}]_{he} \Big\} \\
&\quad \times \Big\{ \delta_{gP} \delta_{iN} [W]_{ij} [C^T]_{jh} + [C]_{gi} [W]_{ij} \delta_{jN} \delta_{hP} \Big\} [C]_{ef} [\mathbf{X}_{f\prime}]_{fc} \\
&\quad + [C^T]_{cd} [U^{-1}]_{de} \delta_{eP} \delta_{fN} [\mathbf{X}_{f\prime}]_{fc} \Bigg]
\end{aligned} \tag{S 35}$$

Where we have used $[C^T] = [\hat{C}]$, $[C] = [\hat{C}^T]$, (S 2), and (S 3) to obtain $\frac{\partial [C^T]_{cd}}{\partial [C^T]_{NP}} = \frac{\partial [\hat{C}]_{cd}}{\partial [\hat{C}]_{NP}} = \delta_{cN} \delta_{dP}$

and $\frac{\partial [C]_{ef}}{\partial [C^T]_{NP}} = \frac{\partial [\hat{C}^T]_{ef}}{\partial [\hat{C}]_{NP}} = \delta_{eP}\delta_{fN}$ in equation (S 35).

Continuing, we expand out the terms:

$$
\begin{aligned}
&\frac{\partial \mathcal{E}(C^T)}{\partial [C^T]_{NP}} \\
&\quad = - [U^{-1}]_{ag}[U^{-1}]_{hb}\delta_{gP}\delta_{iN}[W]_{ij}[C^T]_{jh}[\mathbf{Y}_{f\prime}]_{ba} \\
&\quad - [U^{-1}]_{ag}[U^{-1}]_{hb}[C]_{gi}[W]_{ij}\delta_{jN}\delta_{hP}[\mathbf{Y}_{f\prime}]_{ba} \\
&\quad + \delta_{cN}\delta_{dP}[U^{-1}]_{de}[C]_{ef}[\mathbf{X}_{f\prime}]_{fc} \\
&\quad - [C^T]_{cd}[U^{-1}]_{dg}[U^{-1}]_{he}\delta_{gP}\delta_{iN}[W]_{ij}[C^T]_{jh}[C]_{ef}[\mathbf{X}_{f\prime}]_{fc} \\
&\quad - [C^T]_{cd}[U^{-1}]_{dg}[U^{-1}]_{he}[C]_{gi}[W]_{ij}\delta_{jN}\delta_{hP}[C]_{ef}[\mathbf{X}_{f\prime}]_{fc} \\
&\quad + [C^T]_{cd}[U^{-1}]_{de}\delta_{eP}\delta_{fN}[\mathbf{X}_{f\prime}]_{fc}
\end{aligned} \quad . \tag{S 36}
$$

$$
\begin{aligned}
&= - \delta_{iN}[W]_{ij}[C^T]_{jh}[U^{-1}]_{hb}[\mathbf{Y}_{f\prime}]_{ba}[U^{-1}]_{ag}\delta_{gP} \\
&\quad - [W]_{Ni}[C^T]_{ig}[U^{-1}]^T_{ga}[\mathbf{Y}^T_{f\prime}]_{ab}[(U^{-1})^T]_{bh}\delta_{hP} \\
&\quad + \delta_{cN}[\mathbf{X}^T_{f\prime}]_{cf}[C^T]_{fe}[(U^{-1})^T]_{ed}\delta_{dP} \\
&\quad - [W]_{Nj}[C^T]_{jh}[U^{-1}]_{he}[C]_{ef}[\mathbf{X}_{f\prime}]_{fc}[C^T]_{cd}[U^{-1}]_{dg}\delta_{gP} \\
&\quad - [W]_{Ni}[C^T]_{ig}[(U^{-1})^T]_{gd}[C]_{dc}[\mathbf{X}^T_{f\prime}]_{cf}[C^T]_{fe}[(U^{-1})^T]_{eh}\delta_{hP} \\
&\quad + \delta_{fN}[\mathbf{X}_{f\prime}]_{fc}[C^T]_{cd}[U^{-1}]_{de}\delta_{eP}
\end{aligned} \quad . \tag{S 37}
$$

Where in (S 37), we applied (S 4), (S 5), (S 6), and (S 7) when necessary. For example, $[W]_{ij}\delta_{jN} = [W]_{iN}$. We note that the controllability Gramian matrix W is symmetric, thus taking the transpose has no effect on it: that is, $[W^T]_{iN} = [W]_{Ni} = [W]_{iN}$.

And finally,

$$
\begin{aligned}
&\frac{\partial \mathcal{E}(C^T)}{\partial [C^T]_{NP}} \\
&\quad = - [WC^TU^{-1}\mathbf{Y}_{f\prime}U^{-1}]_{NP} \\
&\quad - [WC^TU^{-1}\mathbf{Y}_{f\prime}U^{-1}]_{NP} \\
&\quad + [\mathbf{X}_{f\prime}C^TU^{-1}]_{NP} \\
&\quad - [WC^TU^{-1}C\mathbf{X}_{f\prime}C^TU^{-1}]_{NP} \\
&\quad - [WC^TU^{-1}C\mathbf{X}_{f\prime}C^TU^{-1}]_{NP} \\
&\quad + [\mathbf{X}_{f\prime}C^TU^{-1}]_{NP}
\end{aligned} \quad . \tag{S 38}
$$

$$
\begin{aligned}
&\therefore \frac{\partial \mathcal{E}(C^T)}{\partial [C^T]_{NP}} \\
&\quad = - 2[WC^TU^{-1}\mathbf{Y}_{f\prime}U^{-1}]_{NP} - 2[WC^TU^{-1}C\mathbf{X}_{f\prime}C^TU^{-1}]_{NP} \\
&\quad + 2[\mathbf{X}_{f\prime}C^TU^{-1}]_{NP} \\
&\quad = - 2WC^T(CWC^T)^{-1}\mathbf{y}_f\mathbf{y}_f^T(CWC^T)^{-1} \\
&\quad - 2WC^T(CWC^T)^{-1}Ce^{At_f}e^{A^Tt_f}C^T(CWC^T)^{-1} \\
&\quad + 2e^{At_f}e^{A^Tt_f}C^T(CWC^T)^{-1}
\end{aligned} \quad . \tag{S 39}
$$

Where in equation (S 38), we again used (S 4), (S 5), (S 6), and (S 7) when necessary. We also note that the output controllability Gramian $U = (CWC^T)$, and constant matrices $\mathbf{X}_{f'} = e^{At_f} e^{A^T t_f}$, $\mathbf{Y}_{f'} = \mathbf{y}_f \mathbf{y}_f^T$ are symmetric. Thus, $(U^{-1})^T = U^{-1}$, $\mathbf{X}_{f'}^T = \mathbf{X}_{f'}$, and $\mathbf{Y}_{f'}^T = \mathbf{Y}_{f'}$.

In equation (S 39), we concatenate the terms and write out the gradient information final form. Thus, the energy gradient information is derived.

5 Selecting C^*_{binary} from C^*

From the main text, two methods were proposed to obtain the sparse binary optimal target node set, C^*_{binary} from the converged dense real matrix C^*. Where by sparse binary, we mean that the $[C^*_{\text{binary}}]_{ij} = 1$ if node j is the i-th target node out of all possible P target nodes, and zero everywhere else. Here, we further expound on the selection schemes using an example of an $N = 9$ stem network with pre-selected $M = 3$ number of driver nodes arranged at nodes 1, 4, and 7. The goal is to use TPGM to find $P = 6$ target nodes which result in reduced control energy. See figure S1.

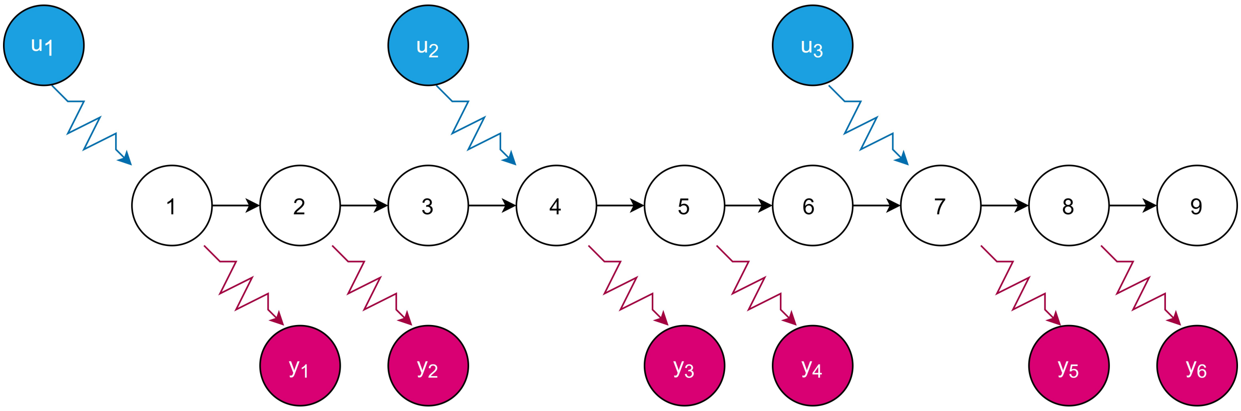

Figure S1: When driver nodes are fixed, the arrangement of which target nodes to be controlled can affect the control energy. From numerical experimentation, the 6 target nodes which results in minimal control energy are nodes {1,2,4,5,6,7}. The optimal target node set has reduced path distance from driver nodes to target nodes, which is consistent with the literature [S5]

In a typical run of TPGM, the algorithm will bring the initial proposed random matrix C_0 towards the direction of quickest decreasing energy, based on the cost function (S 23) and cost function derivative (S 39). A converged solution matrix C^* may look something like figure S2 (left panel).

Based on the description within the main text to choose from each column the largest absolute element, starting with the columns with the largest absolute values, C^*_{binary} can be obtained (figure S2 (right panel)). C^*_{binary} remains numerically similar to the obtained optimal matrix C^* since we are picking the largest element from each column.

Another method described in the main text to choose C^*_{binary} is based on the idea of suppressing C^* insignificant elements and then using the importance index formula (eqn (10) in the main text) to pick P target nodes with the P largest importance index. This is illustrated in figure S3.

Numerically, we can verify that target nodes {1,2,4,5,7,8} are indeed the optimal target nodes, and that the proposed conversion schemes from C^* to C^*_{binary} is indeed effective. Selecting 6 target nodes from all possible 9 nodes in the stem network is a (9 choose 6) $\binom{9}{6} = 84$ combinatorics, which quickly becomes infeasible when N increases. Thus, in a complex network, a brute force search is infeasible.

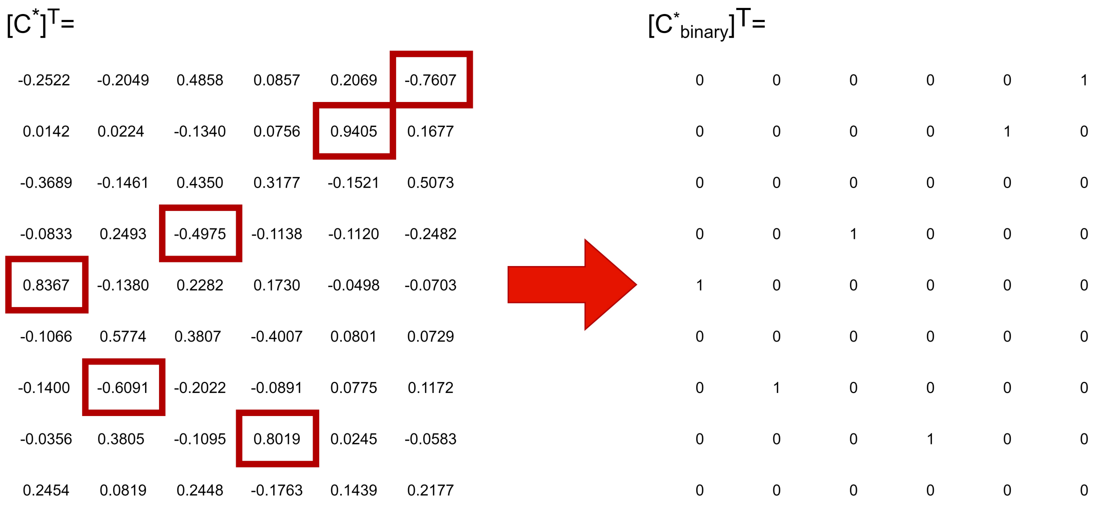

Figure S2: Converting C^* to C^*_{binary} using the largest column absolute value method.

6 Dataset

In the main text Discussion, we said that sometimes the condition number of the Gramian matrix can be infeasibly high, despite increasing the number of driver nodes. To make the condition number feasible, we either normalise the link weights, or set the connection strength to be uniform random $[0.5, 1.5]$ if the connection strength is not specified. Here, we document the network dataset that we used, the source, as well as any necessary information.

Electronic circuits [S6]: the electronic network link weights are unspecified. For our experimentations, we used $\{a_{ij}\} = 1$ if there is a link from node j to node i, and zero otherwise. The dataset is available for download at: http://www.weizmann.ac.il/mcb/UriAlon/.

Food web [S7–S9]: Maspalomas and Rhode networks link weights are specified. However, the condition number of the Gramian can be infeasibly high without normalization. For Maspalomas network, we normalise the link weights by $1e5$, and for Rhode network, we normalise the link weights by $1e4$. StMarks network link weight is unspecified. However, using $\{a_{ij}\} = \{1, 0\}$ will result in the condition number of the Gramian to be high. Thus, for StMarks, we set the link weights to be random uniform $[0, 1.5]$ if $\{a_{ij}\}$ is a nonzero link. The dataset can be downloaded at: http://vlado.fmf.uni-lj.si/pub/networks/data/bio/foodweb/foodweb.htm.

Physicians [S10]: for the physicians networks, the data set represents "person a" trusts "person b." For this reason, similar to ref. [S11], control is more sensible when the connection direction is reversed to reflect that "person a" is influenced by "person b." For the physicians networks, link weight is unspecified. Using binary link weights will result in a high condition number of the Gramian, thus we used random uniform $[0, 1.5]$ in this case. The dataset is available online at: http://moreno.ss.uci.edu/data.html.

Teacher-student [S12]: the link weight is unspecified, and we used binary link weight. The dataset is available online at: http://moreno.ss.uci.edu/data.html.

Highschool [S13]: the link weight is specified with $\{a_{ij}\} = \{0, 1, 2\}$, and we normalised the link weights by 2. The dataset is available online at: http://konect.uni-koblenz.de/networks/moreno_highschool.

6.1 Main text table 1 additional information

Here, we present additional information of standard deviations to the table 1 presented in the main text.

$[C^*]^T =$

0.9545	-0.0076	-0.0486	0.0689	0.0489	0.0593
-0.0786	0.9140	0.0398	-0.0816	0.1343	0.1013
0.0374	-0.2449	0.5303	-0.5548	0.1979	0.0607
0.0237	-0.0519	-0.6854	-0.0043	-0.1314	0.0571
-0.0908	-0.1079	0.3092	0.8030	0.2280	0.0661
0.1992	0.2443	0.3338	0.0416	-0.5822	-0.1793
0.1179	0.1026	-0.1183	-0.0782	0.7237	-0.1445
0.0438	0.0297	0.0710	0.0269	-0.0134	0.9231
0.1290	0.1368	0.1424	0.1658	0.0894	-0.2636

$[C^*_{\text{suppressed}}]^T =$

0.9545	0	0	0	0	0
0	0.9140	0	0	0	0
0	0	0	-0.5548	0	0
0	0	-0.6854	0	0	0
0	0	0	0.8030	0	0
0	0	0	0	-0.5822	0
0	0	0	0	0.7237	0
0	0	0	0	0	0.9231
0	0	0	0	0	0

$[C^*_{\text{binary}}]^T =$

1	0	0	0	0	0
0	1	0	0	0	0
0	0	0	0	0	0
0	0	1	0	0	0
0	0	0	1	0	0
0	0	0	0	0	0
0	0	0	0	1	0
0	0	0	0	0	1
0	0	0	0	0	0

Figure S3: Converting C^* to C^*_{binary} using the suppressed matrix method. The standard deviation, σ, of $|C^*|$ is calculated. We can observe that in the top panel of matrix $[C^*]^T$, directly using eqn (10) in the main text to evaluate the importance index may not allow us to find the correct optimal target nodes due to the presence of insignificant elements affecting the row absolute summation. In this example, we suppress matrix elements $[|C^*|]^T_{ij}$ whose values are $\leq 2.0\,\sigma$ and set those to be zero (middle panel). Then, using the suppressed matrix and evaluating the importance index, the correct optimal target nodes can be found.

References

[S1] Kaare Brandt Petersen et al. The matrix cookbook.

[S2] Guoqi Li, Pei Tang, Ziyang Meng, Changyun Wen, Jing Pei, and Luping Shi. Optimization on matrix manifold based on gradient information and its applications in network control. *Physica A: Statistical Mechanics and its Applications*, 2018.

[S3] Isaac Klickstein, Afroza Shirin, and Francesco Sorrentino. Energy scaling of targeted optimal

Table S1: Additional information on table 1 in the main text. $\sigma[\mathcal{E}(C_0)]$ refers to the standard deviation of the 10 independent initializations matrix energy cost. $\sigma[\mathcal{E}(C^*)]$ is the standard deviation of the 10 independent converged energy cost. $\mu[\mathcal{E}([C^*_{\text{binary}}]^t)]$ is the mean of the 10 obtained binary optimal candidate solutions. $\sigma[\mathcal{E}(C_{rand})]$ is the standard deviation of energy cost needed in 100 independent realizations of random selection of target nodes.

Network	N	edges	$\sigma[\mathcal{E}(C_0)]$	$\sigma[\mathcal{E}(C^*)]$	$\mu[\mathcal{E}([C^*_{\text{binary}}]^t)]$	$\sigma[\mathcal{E}([C^*_{\text{binary}}]^t)]$	$\sigma[\mathcal{E}(C_{rand})]$
**Model**							
SF300	300	750	4.08E07	2.47E03	7.89E06	5.07E06	1.35E08
ER300	300	750	1.03E06	9.93E01	3.12E05	2.01E05	1.33E06
**Electronic circuit**							
Circuit-s838	512	819	1.13E06	1.52E02	4.28E05	4.48E05	2.53E06
Circuit-s420	252	399	6.33E04	1.26E01	7.97E04	1.06E05	2.74E06
Circuit-s208	122	189	1.58E04	7.26	6.76E05	2.07E06	6.21E08
**Food web**							
StMarks	54	356	3.36E03	2.35	4.09E02	9.99E01	3.74E03
Maspalomas	24	82	1.58E03	9.21	6.35E01	1.32E01	2.22E05
Rhode	19	53	3.97E02	0.689	5.06E01	2.06E01	2.24E05
**Social Influence**							
Phys-discuss-rev	231	565	3.82E03	2.23E01	8.21E03	2.74E03	5.93E05
Teacher-student	60	94	3.47E01	0.526	6.76E01	1.82	1.12E02
**Social**							
Phys-friend-rev	228	506	2.29E04	3.02E01	8.42E03	3.41E03	2.63E04
Highschool	70	366	5.34E04	8.84	1.77E03	2.77E03	5.07E04

control of complex networks. *Nature communications*, 8:15145, 2017.

[S4] Donald E Kirk. *Optimal control theory: an introduction*. Courier Corporation, 2012.

[S5] Yu-Zhong Chen, Le-Zhi Wang, Wen-Xu Wang, and Ying-Cheng Lai. Energy scaling and reduction in controlling complex networks. *Royal Society open science*, 3(4):160064, 2016.

[S6] Ron Milo, Shalev Itzkovitz, Nadav Kashtan, Reuven Levitt, Shai Shen-Orr, Inbal Ayzenshtat, Michal Sheffer, and Uri Alon. Superfamilies of evolved and designed networks. *Science*, 303(5663):1538–1542, 2004.

[S7] Daniel Baird, J Luczkovich, and Robert R Christian. Assessment of spatial and temporal variability in ecosystem attributes of the st marks national wildlife refuge, apalachee bay, florida. *Estuarine, Coastal and Shelf Science*, 47(3):329–349, 1998.

[S8] J Almunia, G Basterretxea, J Arıstegui, and RE Ulanowicz. Benthic-pelagic switching in a coastal subtropical lagoon. *Estuarine, Coastal and Shelf Science*, 49(3):363–384, 1999.

[S9] D Correll. Water exchanges in the rhode river watershed, anne arundel county, maryland usa. (Unpublished manuscript) Smithsonian Institute, Chesapeake Bay Center for Environmental Research, Edgewater, Maryland 21037-0028 USA.

[S10] Ronald S Burt. Social contagion and innovation: Cohesion versus structural equivalence. *American journal of Sociology*, 92(6):1287–1335, 1987.

[S11] Justin Ruths and Derek Ruths. Control profiles of complex networks. *Science*, 343(6177):1373–1376, 2014.

[S12] Douglas R White and Karl P Reitz. Rethinking the role concept: Homomorphisms on social networks. *Research methods in social network analysis*, pages 429–488, 1989.

[S13] James Samuel Coleman et al. Introduction to mathematical sociology. *Introduction to mathematical sociology.*, 1964.
